# Feature selection via robust weighted score for high dimensional binary class-imbalanced gene expression data^☆^

**DOI:** 10.1016/j.heliyon.2024.e38547

**Published:** 2024-09-30

**Authors:** Zardad Khan, Amjad Ali, Saeed Aldahmani

**Affiliations:** Department of Statistics and Business Analytics, United Arab Emirates University, Al Ain, United Arab Emirates

**Keywords:** Gene expression data, Unbalanced class distribution, Features selection, Robust score, Support vectors

## Abstract

In this paper, a robust weighted score for unbalanced data (ROWSU) is proposed for selecting the most discriminative features for high dimensional gene expression binary classification with class-imbalance problem. The method addresses one of the most challenging problems of highly skewed class distributions in gene expression datasets that adversely affect the performance of classification algorithms. First, the training dataset is balanced by synthetically generating data points from minority class observations. Second, a minimum subset of genes is selected using a greedy search approach. Third, a novel weighted robust score, where the weights are computed by support vectors, is introduced to obtain a refined set of genes. The highest scoring genes based on this approach are combined with the minimum subset of genes selected by the greedy search approach to form the final set of genes. The novel method ensures the selection of the most discriminative genes, even in the presence of skewed class distribution, thereby improving the performance of the classifiers. The performance of the proposed ROWSU method is evaluated on 7 gene expression datasets. Classification accuracy, sensitivity and F_1_-score are used as performance metrics to compare the proposed ROWSU algorithm with several other state-of-the-art methods. Boxplots and stability plots are also constructed for a better understanding of the results. The results show that the proposed method outperforms the existing feature selection procedures based on classification performance from *k* nearest neighbors (*k*NN) and random forest (RF) classifiers.

## Highlights

• The method addresses skewed class distributions in gene expression datasets, improving classification performance.
• It selects the most discriminative genes despite skewed distributions, enhancing classifier accuracy.
• The method reduces dataset dimensionality, aiding high-dimensional classification tasks.

## 1 Introduction

Gene expression datasets have an important role in enabling researchers to diagnose diseases and investigate the complexities of biological processes. These datasets, generated through deoxyribonucleic acid (DNA) microarrays and ribonucleic acid (RNA) sequencing, provide important insights into gene activities of various organism cells. These datasets comprise a large number of features/genes, many of which are redundant and noisy, and may not play a significant role in diagnosing a specific disease or biological process. To overcome this issue, it is necessary to select a small set of genes that have the most discriminative power to classify the samples into their correct classes (i.e., whether the organisms from which biopsies/tissues are taken have the disease or not). Feature selection has several advantages, such as increasing accuracy and reducing the execution time of classification models. Additionally, feature selection can also reduce the curse of dimensionality and increase the generality of classifiers. There are three main categories of feature selection techniques given as follows:

1. **Embedded methods:** These procedures integrate feature selection within the training process of a machine learning classifier. Examples of the embedded methods are regularization techniques like least absolute shrinkage and selection operator (LASSO) [1], ridge regression [2] and decision tree-based feature importance [3].
2. **Wrapper methods:** This class of feature selection methods computes all possible feature subsets using a specific machine learning algorithm and choose the features for which the fitted model gives high performance. Forward selection [4], [5], backward elimination [4], and recursive feature elimination [6] are the examples of the wrapper methods.
3. **Filter methods:** Filter methods select the most discriminative features by using statistical computations. These procedures determine the association of each feature with the response variable, which is used as a relevance score for the features. The Pearson product-moment correlation, Relief-based algorithms [7], uncorrelated shrunken centroid (USC) [8] and minimum redundancy-maximum relevance (MRMR) [9] algorithms are the examples of filter methods. Other examples of filter methods can be seen in [10], [11], [12], [13], [14], [15]

The above feature selection procedures have numerous advantages across various real-world applications. Selecting the most discriminative features not only simplifies the model but also enhances its performance and interpretability and allowing the researchers to obtain important insights. Moreover, feature selection plays an important role in preventing classifiers from overfitting and enables to generalize the models effectively for new data. However, these procedures fail to perform well when the data has an unbalanced class distribution. Class-imbalanced problem is very common in gene expression datasets, especially when dealing with exceptionally rare diseases [16]. The unbalanced class distribution can skew the features importance which leads to biased selection of features. In such situations, the unbalance problem may cause an over selection of majority class at the expense of minority class. To avoid the issue of skewed class distributions, there are two widely used remedies. The first remedy is balancing the data through oversampling the minority class observations or undersampling the majority class observations which generates a more equitable distribution [17], [18]. The second remedy is to use specialized techniques designed to avoid unbalanced problem such as cost-sensitive learning procedures or ensemble algorithms [19], [20], [21], [22], [23]. These strategies can enhance the models performance and ensure more accurate results in various unbalanced gene expression datasets.

This work proposes a filtering feature selection procedure for unbalanced gene expression datasets. The novel method named as robust weighted score for unbalanced dataset (ROWSU) selects the most relevant genes by using the following steps. In the first step, it balances the dataset through sub-sampling from the minority class to generate new data points by applying the ordinary mean function on the features in the samples. These data points are then added to the original data which makes it balanced. In the second step, a minimum subset of features is chosen through a greedy search approach given in [24]. In the third step, a novel robust weighted score is used to find the final set of genes. The proposed robust weighted score is the hybridization of a novel robust Fisher type score with the corresponding weights computed through support vectors. The highest scoring genes are combined with the minimum subset of genes to get the final set of the most relevant features. The proposed method is designed to achieve an adequate classification performance when the datasets are extremely unbalanced. The performance of the ROWSU algorithm is assessed via 7 benchmark problems. Classification accuracy, sensitivity and F_1_-score are used to evaluate the performance of the proposed method in comparison with the ordinary procedures, i.e., Fisher score (Fish) [25], [26], Wilcoxon rank sum test (Wilc) [27], [28], signal to noise ratio (SNR) [29], proportion overlapping score (POS) [24], maximum relevance-minimum redundancy (MRMR) [9], weighted signal to noise ratio (WSNR) [30] and robust Fisher score [31]. Stability plots and boxplots of the results are also constructed for a visual comparison of the models' performance. The results demonstrate the efficacy of the new algorithm which outperforms the other methods in majority of the cases while using random forest (RF) [32] and *k* nearest neighbors (*k*NN) [33] for classification purpose.

The remainder of the manuscript is organized as follows: Section 2 summarizes the related work and Section 3 gives details of the proposed algorithm. In Section 4, the experimental design and results of the proposed method and other state-of-the-art procedures are discussed. This section also provides a short summary of the considered datasets. Finally, the manuscript is concluded in Section 5.

## 2 Related work

Several methods have been proposed in the literature for feature selection in high dimensional gene expression datasets. Feature selection aims at identifying those genes which have the most discriminative power and increases the performance of the models [34]. Feature selection has a crucial impact on the analysis and reduces the execution time of the models [35], [36]. A relative importance procedure has been proposed in [37], which randomly selects subsets of features from the entire space and grows a large number of trees on each of the subsets. The constructed trees are assessed using a validation set of observations. Finally, features are selected based on which the trees correctly classify the maximum number of sample points into their correct classes. A two-stage grey wolf optimization procedure has been proposed for selecting the most relevant features in high-dimensional data problems [38]. This procedure ensures classification performance with a small set of features and reduces training time of the models. Another method, known as minimum redundancy-maximum relevance (MRMR) has been proposed in the literature [9] to determine the most discriminative features. This procedure attains maximum relevance with the response variable while minimizing redundancy. A minimum redundancy-maximum relevance ensemble (MRMRE) as an extension of the MRMR method is given in [39], which has shown improvements. Another feature selection procedure based on principal component analysis technique has been proposed in [40]. This method selects those features that are more discriminative with minimum component variation. A similar method is given in [41], which uses factor analysis technique instead of the principal component analysis. Statistical tests like t-test and Wilcoxon rank-sum test have also been used to select the most relevant features [27], [42]. The authors in [43] presented a feature selection method which computes gene mask for each feature in the data by using the range of the core interval of gene expressions, ensuring the selection of the more relevant genes in classifying the data points to their correct classes, thus avoiding ambiguity. This method selects a minimum subset of features that unambiguously classify the majority of the training observations to their classes correctly by using the gene masks and overlapping scores via the set covering technique. The minimum subset of features and genes having the smallest overlapping scores are combined in the ultimate set of discriminative features. An extension of [43] has been made in [24], where the core interval of gene expression is computed by using a robust form of dispersion, i.e., the interquartile range. The most relevant genes are identified through the proportion of overlapping observations in each class. Features with smaller POS scores are considered more discriminative and relevant to the target variable. Moreover, the relative dominant classes for all features have also been identified. This associates each of the features with the class for which it has a powerful discriminative capability. Then, a set of the most relevant features is identified through the integration of the minimum subset of feature and POS score giving the final set of genes. Another study, as outlined in [44], contributes to the evolving landscape of multi-label classification, building upon diverse feature selection methodologies and emphasizing the efficacy of specific techniques for improved classification outcomes. For further studies about feature selection, additional resources are available in the literature, such as [45], [46], [47], [48]. These methods have several real-life applications and demonstrate promising results. However, they face issues when dealing with class-imbalance in the data.

Researchers have proposed numerous procedures to address class unbalance problems [49], [50]. These include balancing the data through resampling techniques and then applying feature selection methods [51]. These procedures are shown to significantly enhance the performance of the existing procedures. In [52], the authors introduced a feature selection procedure for imbalanced data based on genetic search. While genetic search methods are known for their effectiveness in achieving good performance, it is important to note that they come with a substantial computational burden. The authors in [53] have proposed a feature selection technique for unbalanced data by using class decomposition. This procedure divides the majority class into smaller sub-classes, then uses the proposed technique to select the discriminative features. Another method, given in [54], introduces a wrapper algorithm addressing class imbalance using a balanced loss function. Similarly, the technique given in [55], proposes an ensemble wrapper procedures with resampling for data balancing. The study given in [56] proposes an adaptive algorithm with BPSO and FCBF for feature selection, including resampling and ensemble classification. Moreover, a method presented in [57], where orthogonal variance decomposition is utilized for feature evaluation, specifically considering feature interactions under defined conditions. In another study [58], a hybrid filter-wrapper method for feature selection on imbalanced datasets was proposed. The technique for order preference by similarity to ideal solution (TOPSIS) was used as a filter to extract informative features, while the Binary Jaya algorithm with a time-varying transfer function served as the wrapper to find the optimal feature subset. In the study published in [59], a new method was used to enhance the size of the data, balance the distribution of classes, and select relevant features to prevent overfitting in ovarian cancer (OC) research. The method involved integrating OC-related gene data, merging datasets, imputing missing values using Iterative Logistic Imputation, and addressing class imbalance with Synthetic Minority Oversampling Technique and Edited Nearest Neighbors. Additionally, feature selection was performed using the Shapley Additive Explanations based on Recursive Feature Elimination Cross-Validation (ShapRFECV) to identify significant EOC subtype features while retaining predefined ones. In another study [60], the researchers focus on the challenge of classifying cancer using gene expression data. The study aimed to address the issues of class imbalance and high dimensionality. To handle the imbalance, the researchers used synthetic oversampling techniques to increase the number of samples in the minority class. Additionally, they employed chi-square (CHiS) and information gain (IG) feature selection methods to deal with the high-dimensional gene expression datasets. Furthermore, the study introduced a new method that combines CHiS and IG to identify the most significant genes. Other methods related to class-imbalance problems can be seen in [61], [62], [63], [64].

Considering the above discussion, we have proposed a feature selection procedure for unbalanced gene expression datasets. The novel method balances the data in the first step and then finds a minimum subset of features by using a greedy search approach in conjunction with a novel robust weighted score. The proposed method thus tries to resolve the issue of class-imbalance while correctly classifying patients to their correct classes based on a small number of genes where the ordinary methods perform poorly in skewed class distribution scenarios.

## 3 The robust weighted score for unbalanced data (ROWSU)

Let L=(X,Y) be a given gene expression dataset, where *X* represents the feature matrix with a total of *n* observations and *p* genes/features, (i.e., $X={\left[e_{ij}\right]}_{n\times p}\in {ℜ}^{n\times p}$, i=1,2,…,n and j=1,2,…,p), and *Y* symbolizes the corresponding response with two-classes, i.e., Y∈(−,+). The total number of sample points (*n*) comprises $n^{-}$ negative and $n^{+}$ positive classes, where the class-distribution is extremely unbalanced, i.e., $n^{-}>>>n^{+}$. To select the most discriminative genes, the following steps will be taken into consideration.

### 3.1 Balancing the data

To balance the given dataset L, $m=n^{-}-n^{+}$ random sub-samples are selected from the minority class (+) each of size $n^{\prime }$, i.e., $X_{s}$, where s=1,2,…,m, since there are *p* features and $n^{\prime }$ observations in each of the sub-samples. Therefore, the mean of the $j^{th}$ gene in the $s^{th}$ sample can be calculated as follows:

$${\overset{¯}{G}}_{sj}=\frac{\sum_{i=1}^{n^{\prime }}e_{ij}}{n^{\prime }},$$

where j=1,2,…,p and s=1,2,…,m. In this way, new observations are generated to increase the size of the minority class (+), i.e.,

$${\overset{¯}{X}}_{s}={\left[{\overset{¯}{G}}_{s1},{\overset{¯}{G}}_{s2},\ldots ,{\overset{¯}{G}}_{sp}\right]}_{1\times p},$$

where s=1,2,…,m. The generated data points can be written in a matrix form as follows:

$$\overset{¯}{X}=\left[\begin{array}{c} {\overset{¯}{X}}_{1}\\ {\overset{¯}{X}}_{2}\\ \vdots \\ {\overset{¯}{X}}_{m} \end{array}\right],$$

$$\Rightarrow \overset{¯}{X}=\left[\begin{array}{cccc} {\overset{¯}{G}}_{1\times 1}\; & {\overset{¯}{G}}_{1\times 2}\; & \ldots \; & {\overset{¯}{G}}_{1\times p}\\ {\overset{¯}{G}}_{2\times 1}\; & {\overset{¯}{G}}_{2\times 2}\; & \ldots \; & {\overset{¯}{G}}_{2\times p}\\ \vdots \; & \vdots \; & \ddots \; & \vdots \\ {\overset{¯}{G}}_{m\times 1}\; & {\overset{¯}{G}}_{m\times 2}\; & \ldots \; & {\overset{¯}{G}}_{m\times p} \end{array}\right].$$

Now, write the generated data points with their class label (+), i.e.,

$$L^{\prime }=(\overset{¯}{X},Y=+).$$

Combine the generated data $L^{\prime }$ and the given training data L, i.e.,

$$L^{⁎}=L\cup L^{\prime }.$$

Data $L^{⁎}$ is balanced, consisting of a total of $n^{⁎}$ sample points, where each class has the same number of observations, i.e.,

$$n^{⁎}=n+m,$$

$$\Rightarrow n^{⁎}=(n^{-}+n^{+})+(n^{-}-n^{+}),$$

$$\Rightarrow n^{⁎}=2n^{-}.$$

In $L^{⁎}$, there are $n^{-}$ data points of each class. Use $L^{⁎}$ instead of the original data L to select the most discriminative features.

### 3.2 Selecting minimum subset of features

To select a minimum subset of $p^{\prime }$ features from the balanced data $L^{⁎}$ given in Subsection 3.1, this work has used the greedy search approach given in [24]. The selected subset of $p^{\prime }$ features must accurately classify the majority of the data points in the training dataset while averting the impact of outliers in gene expression values. The selection process uses gene masks and proportion overlapping scores (POS) [24]. Initially, the gene with the maximum number of 1-bits in its mask is included in the subset. If there are multiple genes with the same number of 1-bits, the gene with the smallest POS score is added to the subset. Subsequently, the logical operator *AND* updates the gene masks of the remaining genes to select the next one. Repeat this process until a specified number of genes is reached or no gene has 1-bit in its gene mask. For further discussion on the greedy search approach, refer to [24]. For simplicity, some notations are provided as follows:

${L^{⁎}}_{min}$: Dataset having minimum subset of $p^{\prime }$ features only.

${L^{⁎}}_{Rem}$: Dataset without minimum subset of genes, i.e., having $p^{\prime \prime }=p-p^{\prime }$ features.

### 3.3 Robust Fisher score

In this work a robust Fisher (RFish) score is proposed for binary class problems (standard Fisher score can be seen in [25] and [26]). The proposed RFish score is given in the following equation:

(1) $$\psi_{j}=\frac{n^{+}|{\overset{˜}{\mu }}_{j}^{+}-{\overset{˜}{\mu }}_{j}|+n^{-}|{\overset{˜}{\mu }}_{j}^{-}-{\overset{˜}{\mu }}_{j}|}{n^{+}\Delta_{j}^{+}+n^{-}\Delta_{j}^{-}},\;\text{where,}\;j=1,2,\ldots ,p^{\prime \prime },$$

where $\psi_{j}$ shows robust fisher score for $j^{th}$ feature, ${\overset{˜}{\mu }}_{j}^{+}$ and ${\overset{˜}{\mu }}_{j}^{-}$ are the medians of $j^{th}$ gene for class + and −, respectively. ${\overset{˜}{\mu }}_{j}$ is the global median for $j^{th}$ gene in the given data. Furthermore, $\Delta_{j}^{+}$ and $\Delta_{j}^{-}$ are the mean absolute deviations of $j^{th}$ gene for class + and −, respectively.

Since, the data $L^{⁎}$ has a balanced class distribution, i.e., both classes have $n^{-}$ sample points. Therefore, Equation (1) can be written as follows:

(2) $$\psi_{j}=\frac{\left|{\overset{˜}{\mu }}_{j}^{+}-{\overset{˜}{\mu }}_{j}|+|{\overset{˜}{\mu }}_{j}^{-}-{\overset{˜}{\mu }}_{j}\right|}{\Delta_{j}^{+}+\Delta_{j}^{-}},\;\text{where,}\;j=1,2,\ldots ,p^{\prime \prime }.$$

Using Equation (2), the following set of robust Fisher scores is obtained for all the features in the ${L^{⁎}}_{Rem}$, i.e.,

(3) $$\psi =\{\psi_{1},\psi_{2},\ldots ,\psi_{p^{\prime \prime }}\}.$$

### 3.4 Feature weights (*w*)

For weighting the above score to increase its discriminating ability, the notion of support vectors is exploited [65], [66]. This method uses support vectors to determine an optimal hyperplane that classifies the observations into their correct classes. The hyperplane can be expressed as follows:

(4) $$H:w^{T}.(M(x_{i}))+b,$$

where *w* shows the normal vector to the hyperplane, $x_{i}$ stands for the data point and *b* represents the bias term, while $M(x_{i})$ maps $x_{i}$ into a higher dimensional space.

The distance between the hyperplane H provided in Equation (4) and a specific sample point $x_{i}$ is given by the following formula:

(5) $$\delta_{H}(M(x_{i}))=\frac{\left|w^{T}.(M(x_{i}))+b\right|}{||w|{|}_{2}},$$

where $||w|{|}_{2}$ shows Euclidean norm expressed as:

$$||w|{|}_{2}=\sqrt{w_{1}^{2}+w_{2}^{2}+\ldots +w_{p}^{2}}.$$

The weight vector *w* of features is determined by solving an optimization problem which maximize the distance $\delta_{H}(x_{i})$ (i.e., Equation (5)). The objective function is given by:

$$\min\frac{1}{2}||w|{|}_{2},\;\text{subject to the constraint}\;y_{i}(w^{T}.M(x_{i})+b)\geq 1.$$

For the above optimization problem, the weight and *w* is computed as

$$w=\sum_{i=1}^{n^{⁎}}\alpha_{i}y_{i}x_{i},\text{ where }\alpha_{i}\text{ are Lagrange's multipliers,}$$

(6) $$\Rightarrow w=\{w_{1},w_{2},\ldots ,w_{p^{\prime \prime }}\}.$$

### 3.5 The robust weighted score

The robust weighted score ($\phi_{j}$) for the $j^{th}$ feature is obtained by multiplying the RFish scores given in Equations (3) and feature weights computed in Equation (6), i.e.,

$$\phi_{j}=|w_{j}.\psi_{j}|\;\text{where}\;j=1,2,\ldots ,p^{\prime \prime },$$

(7) $$\Rightarrow \phi =\{\phi_{1},\phi_{2},\ldots ,\phi_{p^{\prime \prime }}\}.$$

Arrange the weights *ϕ* computed in Equation (7) in descending order and select the $p^{\prime ⁎}$ features with the highest score. Denote the data with the selected features by ${L^{⁎}}_{Row}$.

### 3.6 Final set of the most discriminative features

Combine the minimum subset of genes selected by the greedy search approach in Subsection 3.2 and features selected by the Robust Weighted Score in Subsection 3.5, i.e., $p^{⁎}=p^{\prime }+p^{\prime ⁎}$ and the final data becomes:

$${L^{⁎}}_{Final}={L^{⁎}}_{Min}\cup {L^{⁎}}_{Row}.$$

${L^{⁎}}_{Final}$ will be used for model fitting to predict unseen data points.

### 3.7 Algorithm

The steps taken by the proposed ROWSU algorithm are given as follows:

1. Consider a training dataset with an unbalanced class distribution.
2. Make the data balanced by using the technique proposed in Subsection 3.1.
3. Select the most discriminative minimum subset of $p^{\prime }$ features by the greedy search approach given in [24].
4. Select $p^{\prime ⁎}$ features from the remaining data $L_{Rem}^{⁎}$ by using the proposed robust weighted score presented in Subsection 3.5.
5. Combine the selected features in Steps 3 and 4 to get the final data $L_{Final}^{⁎}$ consisting of the most discriminative features.

**Figure 1:**
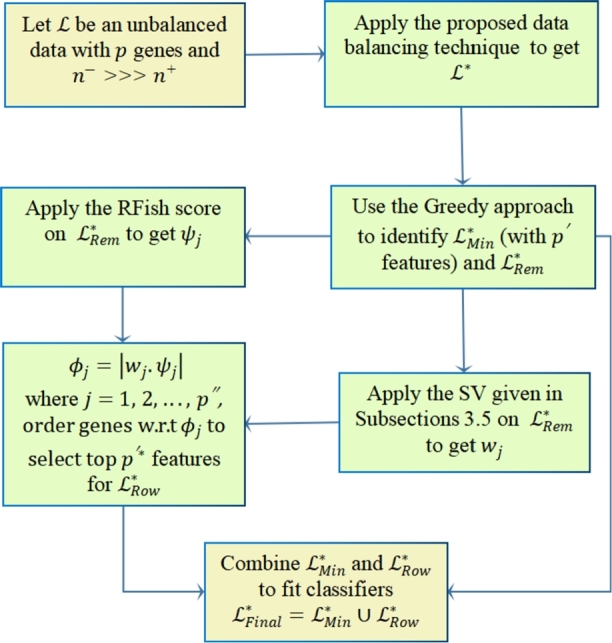
Flowchart of the proposed ROWSU algorithm.

Pseudo-code of the novel ROWSU method is provided in Algorithm 1, and its flow chart in Fig. 1.

**Algorithm 1:**
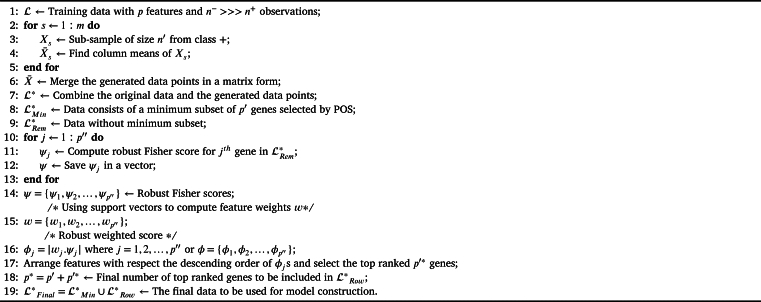
Pseudo code of the novel ROWSU procedure.

## 4 Experiments and results

This section presents the experimental design, analyzed benchmark datasets, evaluation metrics and results of the proposed study. The results are demonstrated in a planned manner, which clarifies the outcomes of the analysis. Further details about the experimental setup and findings of the study are given in the coming subsections.

### 4.1 Benchmark datasets

To evaluate the performance of the proposed method and other existing procedures, 7 benchmark gene expression datasets are used. The considered datasets are briefly summarized in Table 1. The First column of the table represents data ID, the second column shows name of the data, while the third and fourth columns exhibit the number of observations *n* and the number of features *p*, respectively. Class-wise distribution (−,+) is given in the fifth column, while the final column provides the data source.

**Table 1:** Summary of the benchmark datasets.

ID	Data	*p*	*n*	(−,+)	Sources
*D*_1_	DSRBCT	2308	63	(43, 20)	https://tibshirani.su.domains/PAM/Rdist/khan.txt
*D*_2_	DLBCL	5469	77	(58, 19)	https://www.openml.org/search?type=data&status=active&id=45088
*D*_3_	Leukemia	7129	72	(47, 25)	https://www.openml.org/search?type=data&status=active&id=1104
*D*_4_	APEP	10936	130	(69, 61)	https://www.openml.org/search?type=data&status=active&id=1141
*D*_5_	APOU	10936	201	(124, 77)	https://www.openml.org/search?type=data&status=active&id=1124
*D*_6_	Breast	4948	78	(34, 44)	[67]
*D*_7_	Colon	2000	62	(40, 22)	https://www.openml.org/search?type=data&status=active&id=45087

### 4.2 Experimental setup

The experimental design of the study is described in this section. For models assessment, 7 benchmark gene expression datasets have been analyzed. As the proposed method is designed for unbalanced gene expression datasets, therefore the observations are used in a 4:1 ratio, i.e., 80% belonging to negative (−) class and 20% belonging to positive (+) class. This is done by discarding minority class observations randomly, if the imbalanced ratio was not 4:1 in the original data. Each dataset is randomly divided into two groups, i.e., 80% training and 20% testing. The training part is used for model fitting, while evaluation is carried out on the testing part of the data. This splitting process is repeated 500 times for feature selection and classifiers construction. Fisher score (Fish), Wilcoxon rank sum test (Wilc), signal to noise ratio (SNR), proportion overlapping (POS), maximum relevancy-minimum redundancy (MRMR), weighted signal to noise ratio (WSNR) and robust Fisher score (RFish) are used for comparison purposes. Moreover, random forest (RF) and *k* nearest neighbors (*k*NN) have been used as classifiers based on the selected genes. Classification accuracy, sensitivity and F_1_-score are the considered evaluation metrics. R programming is used for the experiments.

Furthermore, different number of the most discriminative features have been selected through the proposed method and the other competitors (i.e., $p^{⁎}=5,10,15,20,25,30$). These sets are used for fitting the considered classifiers to evaluate the performance of the proposed algorithm.

### 4.3 Results

This section presents the results obtained from the feature selection algorithms, including the proposed ROWSU, Fish, Wilc, SNR, POS MRMR, WSNR and RFish, along with the two classifiers random forest (RF) and *k* nearest neighbors (*k*NN) on the $D_{1}$, $D_{2}$, $D_{3}$, $D_{4}$, $D_{5}$, $D_{6}$ and $D_{7}$ datasets. The performance metrics, accuracy, sensitivity and F_1_-score are computed for various values of the selected features $p^{⁎}$.

The results for the $D_{1}$ dataset with varying numbers of features ($p^{⁎}$) are presented in Table 2. The innovative ROWSU procedure consistently outperforms other feature selection methods. In the case of random forest (RF), ROWSU achieves classification accuracy ranging from 94.3% to 97.1%, sensitivity from 77.2% to 86.6%, and F_1_-score from 80.8% to 97.8%. Notably, ROWSU achieves the highest accuracy of 97.1%, sensitivity of 86.6%, and F_1_-score of 97.8% at $p^{⁎}=20$, demonstrating the efficacy of the proposed method. The Fish procedure via RF achieves comparable accuracy and sensitivity for some values of $p^{⁎}$, it does not surpass ROWSU. In the case of *k* nearest neighbors (*k*NN), the ROWSU method outperforms the other techniques with accuracy ranging from 97.1% to 99.4%, sensitivity from 88.6% to 98.5%, and F_1_-score from 96.2% to 99.7%. ROWSU reaches its highest accuracy of 99.4%, sensitivity of 98.5% and F_1_-score of 99.7% at $p^{⁎}=20$. The Fish method also demonstrates comparable performance via RF but falls slightly short of the proposed procedure. Other procedures do not perform satisfactorily via any of the classifiers.

**Table 2:** Results calculated for the top-ranked *p*^⁎^ feature selected by the proposed method and other state-of-the-art procedures using the *D*_1_ dataset.

Metric	$p^{⁎}$	RF								*k*NN							
		ROWSU	Fish	Wilc	SNR	POS	MRMR	WSNR	RFish	ROWSU	Fish	Wilc	SNR	POS	MRMR	WSNR	RFish
Accuracy	5	**0.943**	0.942	0.771	0.796	0.926	0.930	0.933	0.941	**0.971**	0.969	0.761	0.774	0.953	0.953	0.957	0.953
	10	**0.949**	0.946	0.807	0.791	0.932	0.938	0.939	0.936	**0.974**	0.968	0.788	0.775	0.963	0.930	0.970	0.971
	15	**0.967**	0.960	0.822	0.822	0.955	0.951	0.957	0.956	**0.984**	0.977	0.796	0.807	0.981	0.983	0.963	0.977
	20	**0.971**	0.967	0.813	0.811	0.964	0.963	0.940	0.958	**0.994**	0.983	0.819	0.833	0.972	0.984	0.977	0.983
	25	**0.955**	0.945	0.817	0.814	0.947	0.935	0.934	0.948	**0.993**	0.981	0.799	0.804	0.982	0.977	0.981	0.982
	30	**0.964**	0.954	0.812	0.813	0.949	0.941	0.940	0.949	**0.993**	0.986	0.84	0.814	0.978	0.981	0.984	0.943

Sensitivity	5	**0.772**	0.723	0.201	0.196	0.720	0.656	0.682	0.752	**0.886**	0.880	0.180	0.227	0.841	0.774	0.810	0.825
	10	**0.816**	0.794	0.276	0.144	0.808	0.770	0.775	0.812	**0.940**	0.922	0.227	0.209	0.938	0.896	0.928	0.900
	15	**0.842**	0.829	0.151	0.155	0.809	0.769	0.806	0.798	**0.952**	0.949	0.192	0.224	0.916	0.928	0.948	0.882
	20	**0.866**	0.864	0.157	0.176	0.834	0.837	0.785	0.865	**0.985**	0.935	0.236	0.301	0.890	0.937	0.931	0.912
	25	**0.800**	0.770	0.225	0.208	0.784	0.722	0.765	0.759	**0.982**	0.919	0.200	0.261	0.920	0.919	0.936	0.953
	30	**0.852**	0.836	0.136	0.123	0.803	0.761	0.750	0.839	**0.976**	0.957	0.308	0.235	0.925	0.938	0.951	0.956

F_1_-Score	5	**0.808**	0.799	0.556	0.797	0.805	0.780	0.800	0.773	**0.962**	0.874	0.647	0.912	0.881	0.897	0.955	0.936
	10	**0.818**	0.796	0.801	0.808	0.808	0.796	0.809	0.805	**0.961**	0.933	0.682	0.923	0.952	0.929	0.956	0.925
	15	**0.941**	0.909	0.596	0.967	0.933	0.894	0.852	0.909	**0.979**	0.956	0.744	0.975	0.967	0.945	0.941	0.961
	20	**0.943**	0.920	0.688	0.951	0.926	0.926	0.897	0.894	**0.997**	0.967	0.714	0.985	0.989	0.985	0.959	0.965
	25	**0.969**	0.945	0.658	0.929	0.879	0.918	0.857	0.935	**0.982**	0.954	0.645	0.971	0.936	0.933	0.962	0.927
	30	**0.978**	0.952	0.733	0.983	0.944	0.928	0.937	0.969	**0.985**	0.968	0.787	0.975	0.983	0.977	0.965	0.953

Table 3 presents the results obtained for the $D_{2}$ dataset with varying numbers of features ($p^{⁎}$). The novel ROWSU method consistently outperforms other feature selection techniques. When used with the random forest (RF) classifier, ROWSU achieves classification accuracy ranging from 80.3% to 89.4%, sensitivity from 41.2% to 72.8%, and F_1_-score from 81.7% to 90.8%. Notably, ROWSU achieves the highest accuracy of 89.4%, sensitivity of 72.8% and F_1_-score of 90.8% at $p^{⁎}=30$, demonstrating the effectiveness of the proposed method. The Fish, POS and WSNR procedures in RF classification attain similar accuracy and sensitivity for certain values of $p^{⁎}$, but fall short of surpassing ROWSU. In the case of the *k* nearest neighbors (*k*NN) classifier, the ROWSU method outperforms the other techniques, with accuracy ranging from 81.9% to 89.1%, sensitivity from 71.7% to 93.1% and F_1_-score from 78.1% to 91.7%. ROWSU achieves its highest accuracy of 89.1%, sensitivity of 93.1% and F_1_-score of 91.7% at $p^{⁎}=25$. The Fish, WSNR and RFish methods also deliver comparable performance with *k*NN but lag slightly behind the proposed procedure. The remaining procedures do not perform well with any of the classifiers, consistently displaying lower accuracy, sensitivity and F_1_-scores across different values of $p^{⁎}$.

**Table 3:** Results calculated for the top-ranked *p*^⁎^ feature selected by the proposed method and other state-of-the-art procedures using the *D*_2_ dataset.

Metric	$p^{⁎}$	RF								*k*NN							
		ROWSU	Fish	Wilc	SNR	POS	MRMR	WSNR	RFish	ROWSU	Fish	Wilc	SNR	POS	MRMR	WSNR	RFish
Accuracy	5	0.803	0.786	0.768	0.767	**0.815**	0.811	0.801	0.812	0.819	**0.843**	0.759	0.751	0.818	0.825	0.834	0.826
	10	**0.844**	0.814	0.788	0.780	0.816	0.823	0.833	0.831	**0.860**	0.837	0.770	0.775	0.824	0.812	0.856	0.854
	15	**0.870**	0.848	0.816	0.816	0.841	0.862	0.835	0.848	**0.863**	0.830	0.771	0.799	0.842	0.833	0.858	0.827
	20	**0.857**	0.830	0.777	0.792	0.833	0.836	0.854	0.836	**0.877**	0.830	0.773	0.776	0.831	0.826	0.854	0.851
	25	**0.885**	0.826	0.781	0.780	0.827	0.841	0.853	0.859	**0.862**	0.828	0.781	0.788	0.852	0.848	0.854	0.859
	30	**0.894**	0.830	0.801	0.806	0.858	0.856	0.849	0.876	**0.891**	0.844	0.805	0.774	0.848	0.864	0.879	0.889

Sensitivity	5	0.412	0.376	0.221	0.195	0.385	0.358	**0.486**	0.481	**0.717**	0.658	0.274	0.176	0.527	0.610	0.613	0.637
	10	**0.546**	0.448	0.143	0.201	0.389	0.405	0.510	0.473	**0.802**	0.639	0.278	0.354	0.553	0.570	0.716	0.712
	15	**0.615**	0.527	0.235	0.227	0.506	0.481	0.552	0.587	**0.848**	0.638	0.422	0.492	0.624	0.692	0.777	0.795
	20	**0.582**	0.490	0.209	0.218	0.471	0.448	0.568	0.539	**0.813**	0.645	0.399	0.361	0.581	0.619	0.798	0.785
	25	**0.709**	0.565	0.208	0.246	0.516	0.511	0.561	0.621	**0.931**	0.747	0.519	0.563	0.665	0.765	0.760	0.804
	30	**0.728**	0.581	0.260	0.272	0.617	0.539	0.565	0.644	**0.907**	0.742	0.478	0.452	0.618	0.745	0.823	0.819

F_1_-Score	5	**0.817**	0.751	0.510	0.782	0.708	0.664	0.803	0.812	0.781	0.811	0.570	0.758	0.732	0.732	**0.884**	0.846
	10	**0.888**	0.695	0.733	0.858	0.848	0.718	0.867	0.859	**0.915**	0.745	0.601	0.835	0.826	0.648	0.879	0.891
	15	**0.886**	0.664	0.662	0.827	0.694	0.721	0.844	0.878	**0.894**	0.663	0.561	0.765	0.631	0.635	0.884	0.874
	20	**0.876**	0.749	0.539	0.800	0.715	0.681	0.809	0.827	**0.899**	0.689	0.766	0.772	0.719	0.680	0.827	0.894
	25	**0.889**	0.661	0.667	0.789	0.634	0.692	0.879	0.873	**0.917**	0.704	0.629	0.842	0.644	0.733	0.897	0.803
	30	**0.908**	0.742	0.778	0.874	0.727	0.745	0.856	0.895	**0.889**	0.887	0.593	0.815	0.769	0.761	0.871	0.878

Table 4 demonstrates the results computed for the $D_{3}$ dataset. In terms of accuracy, the novel ROWSU method consistently outperforms other techniques for various numbers of features ($p^{⁎}$) for both classifiers. ROWSU shows promising values of accuracy, ranging from 92.0% to 93.9% for RF and 92.5% to 94.0% for *k*NN. In terms of sensitivity, the proposed ROWSU gives better performance than the others. It gives the highest sensitivity ranging from 75.9% to 80.3% for RF and 72.8% to 82.8% for *k*NN. Similarly, highest F_1_-score given by the proposed method ranging from 90.5% to 95.8% for RF and 89.1% to 94.5% for *k*NN. POS and Fish also gives comparable results to the proposed method.

**Table 4:** Results calculated for the top-ranked *p*^⁎^ feature selected by the proposed method and other state-of-the-art procedures using the *D*_3_ dataset.

Metric	$p^{⁎}$	RF								*k*NN							
		ROWSU	Fish	Wilc	SNR	POS	MRMR	WSNR	RFish	ROWSU	Fish	Wilc	SNR	POS	MRMR	WSNR	RFish
Accuracy	5	**0.934**	0.924	0.791	0.781	0.920	0.884	0.905	0.913	**0.927**	0.904	0.781	0.777	0.920	0.866	0.884	0.879
	10	**0.922**	0.917	0.785	0.770	0.915	0.883	0.899	0.910	**0.936**	0.914	0.765	0.774	0.913	0.890	0.901	0.888
	15	**0.938**	0.928	0.789	0.793	0.924	0.885	0.893	0.919	**0.940**	0.897	0.773	0.779	0.919	0.882	0.901	0.895
	20	**0.920**	0.907	0.786	0.781	0.917	0.880	0.904	0.917	**0.925**	0.898	0.763	0.759	0.915	0.889	0.898	0.864
	25	**0.930**	0.919	0.800	0.805	0.923	0.889	0.900	0.925	**0.933**	0.905	0.784	0.787	0.925	0.898	0.904	0.900
	30	**0.939**	0.916	0.808	0.798	0.927	0.889	0.902	0.896	**0.935**	0.892	0.801	0.799	0.926	0.910	0.895	0.917

Sensitivity	5	**0.792**	0.769	0.181	0.129	0.740	0.575	0.663	0.679	**0.754**	0.662	0.239	0.165	0.749	0.408	0.544	0.573
	10	**0.760**	0.759	0.123	0.136	0.720	0.597	0.673	0.683	**0.828**	0.652	0.225	0.289	0.638	0.578	0.628	0.635
	15	**0.803**	0.756	0.101	0.138	0.731	0.532	0.631	0.689	**0.797**	0.532	0.203	0.247	0.634	0.453	0.541	0.657
	20	**0.770**	0.730	0.120	0.093	0.741	0.577	0.624	0.658	**0.776**	0.575	0.224	0.220	0.669	0.554	0.514	0.677
	25	**0.759**	0.698	0.094	0.119	0.716	0.568	0.634	0.669	**0.728**	0.542	0.244	0.257	0.656	0.536	0.572	0.658
	30	**0.782**	0.683	0.098	0.075	0.713	0.553	0.582	0.679	**0.756**	0.502	0.264	0.236	0.689	0.598	0.517	0.680

F_1_-Score	5	**0.905**	0.903	0.786	0.891	0.923	0.882	0.886	0.875	**0.932**	0.900	0.777	0.918	0.925	0.882	0.919	0.924
	10	**0.941**	0.909	0.809	0.911	0.923	0.873	0.933	0.899	**0.939**	0.891	0.773	0.923	0.900	0.891	0.928	0.937
	15	**0.953**	0.877	0.723	0.905	0.886	0.850	0.926	0.924	0.891	0.845	0.714	0.900	0.864	0.845	0.904	**0.916**
	20	**0.958**	0.900	0.809	0.918	0.900	0.877	0.952	0.928	**0.923**	0.873	0.768	0.927	0.905	0.891	0.911	0.922
	25	**0.947**	0.914	0.809	0.932	0.914	0.905	0.935	0.937	**0.935**	0.877	0.745	0.932	0.914	0.891	0.918	**0.935**
	30	**0.955**	0.927	0.791	**0.955**	**0.955**	0.923	0.926	0.948	**0.945**	0.914	0.777	0.914	0.927	0.914	0.927	0.926

Table 5 demonstrates a detailed comparison of the novel method selecting various number ($p^{⁎}$) of features in comparison with the existing procedures on the $D_{4}$ dataset. The ROWSU method gives comparable performance to the MRMR in terms of accuracy vis both RF and *k*NN classifications. In terms of sensitivity, the proposed method gives comparable performance to the existing procedures, i.e., Fish, POS and MRMR. It is evident from the table that the proposed method outperforms the classical procedures in the majority of the situations. It consistently shows competitive performance for different $p^{⁎}$ values.

**Table 5:** Results calculated for the top-ranked *p*^⁎^ feature selected by the proposed method and other state-of-the-art procedures using the *D*_4_ dataset.

Metric	$p^{⁎}$	RF								*k*NN							
		ROWSU	Fish	Wilc	SNR	POS	MRMR	WSNR	RFish	ROWSU	Fish	Wilc	SNR	POS	MRMR	WSNR	RFish
Accuracy	5	0.986	0.983	0.858	0.896	0.991	0.993	0.973	**0.999**	**0.985**	0.971	0.835	0.855	0.982	0.978	0.955	0.980
	10	**0.998**	0.987	0.921	0.919	0.993	0.997	0.987	0.991	**1.000**	0.984	0.876	0.871	0.988	0.993	0.976	0.998
	15	**0.998**	0.990	0.946	0.944	0.991	**0.998**	0.986	**0.998**	**0.999**	0.995	0.897	0.900	0.989	0.996	0.985	0.981
	20	**0.999**	0.995	0.938	0.950	0.990	**0.999**	0.988	**0.999**	**0.996**	0.995	0.898	0.890	0.990	**0.996**	0.983	0.985
	25	**0.999**	0.996	0.962	0.956	0.990	0.997	0.992	0.990	**0.995**	0.993	0.908	0.894	0.990	**0.995**	0.985	0.989
	30	**0.998**	0.996	0.959	0.961	0.987	**0.998**	0.990	0.987	0.993	**0.996**	0.899	0.890	0.982	**0.996**	0.989	0.990

Sensitivity	5	0.981	0.960	0.533	0.651	**1.000**	0.982	0.962	0.995	0.957	0.917	0.406	0.474	**0.987**	0.953	0.898	0.987
	10	0.995	0.979	0.782	0.729	**0.998**	0.996	0.994	**0.998**	**1.000**	0.968	0.548	0.539	0.983	**1.000**	0.955	0.980
	15	**1.000**	0.995	0.808	0.781	0.996	0.998	0.996	**1.000**	0.993	**1.000**	0.557	0.584	0.996	**1.000**	0.987	0.984
	20	**1.000**	**1.000**	0.767	0.813	0.992	**1.000**	**1.000**	**1.000**	0.978	0.995	0.574	0.602	**1.000**	**1.000**	0.990	0.990
	25	**1.000**	**1.000**	0.841	0.845	**1.000**	**1.000**	**1.000**	0.995	0.975	0.987	0.624	0.647	**1.000**	0.995	**1.000**	0.982
	30	0.996	**1.000**	0.849	0.872	0.988	**1.000**	**1.000**	**1.000**	0.968	0.994	0.632	0.575	0.993	**0.996**	0.993	0.998

F_1_-Score	5	0.989	0.948	0.652	0.953	0.963	0.988	0.985	0.988	**0.989**	0.914	0.598	0.959	0.931	0.958	0.975	0.985
	10	0.988	0.978	0.773	0.925	**0.989**	**0.989**	0.980	**0.989**	**1.000**	0.960	0.712	0.967	0.978	0.960	0.987	0.980
	15	**0.993**	0.985	0.843	0.988	0.990	0.989	0.991	0.989	**0.999**	**0.999**	0.766	0.988	0.963	**0.999**	0.991	0.997
	20	**0.995**	0.986	0.854	0.968	0.983	0.986	0.992	**0.995**	0.990	**0.993**	0.672	0.978	0.969	0.988	0.992	0.992
	25	0.993	0.966	0.882	0.946	0.953	0.983	**0.995**	0.994	0.990	0.993	0.740	0.942	0.958	**0.999**	0.997	0.986
	30	**0.999**	**0.999**	0.970	0.985	0.985	0.989	0.986	**0.999**	0.972	0.986	0.816	0.955	0.967	**0.999**	0.995	0.996

**Figure 2:**
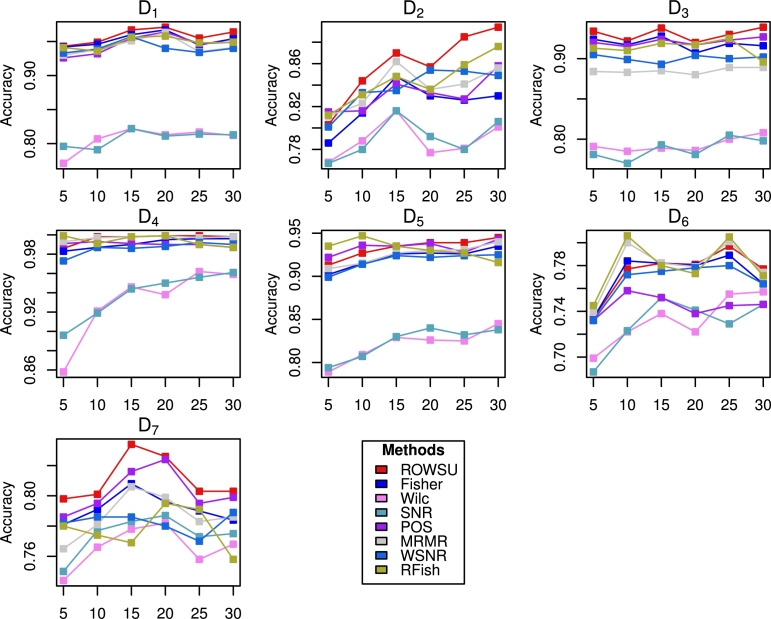
Accuracy computed for the top ranked features selected by the proposed method and other state-of-the-art procedures using RF.

**Figure 3:**
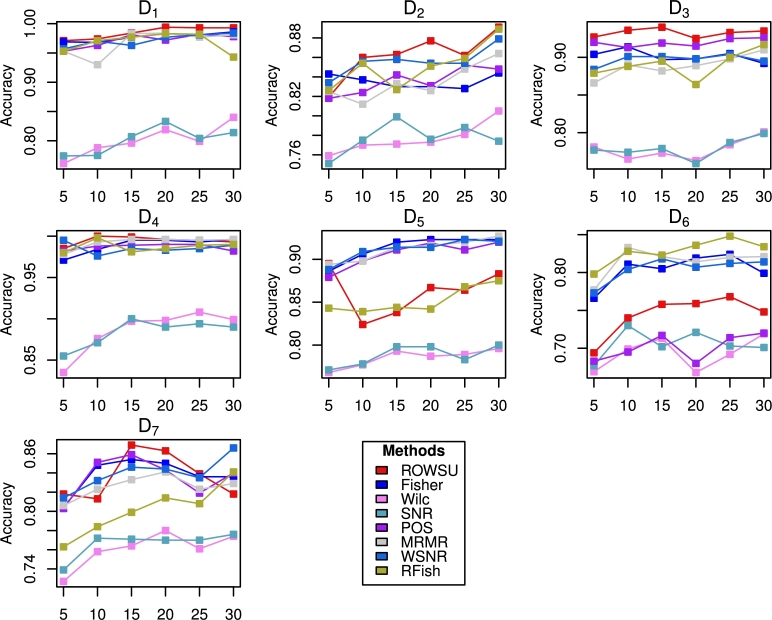
Accuracy computed for the top ranked features selected by the proposed method and other state-of-the-art procedures using *k*NN.

**Figure 4:**
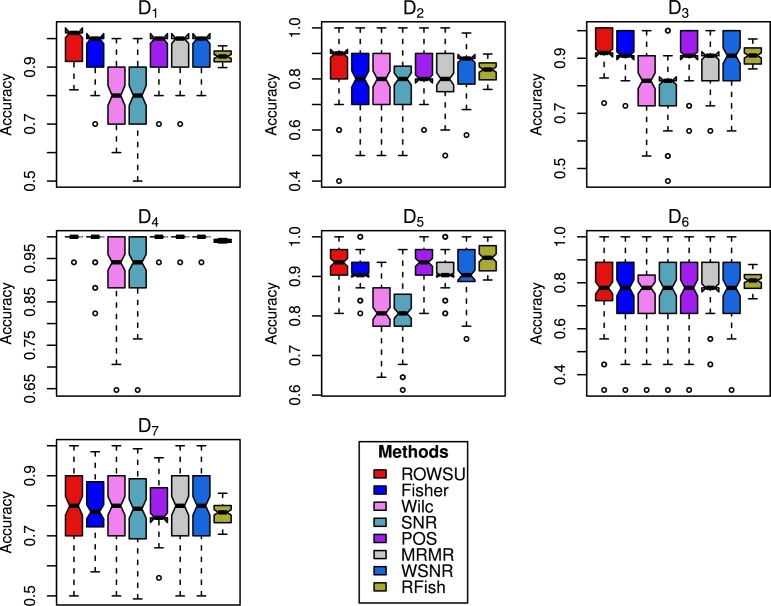
Accuracy computed for the top 10 features by the proposed method and other state-of-the-art procedures using RF.

**Figure 5:**
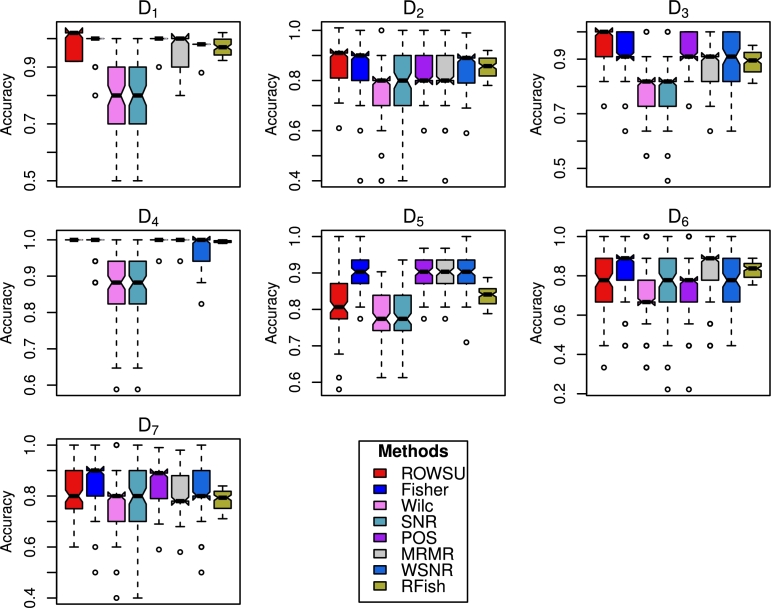
Accuracy computed for the top 10 features by the proposed method and other state-of-the-art procedures using *k*NN.

The findings based on $D_{5}$, $D_{6}$ and $D_{7}$ datasets lead to similar conclusions. The results for these datasets are presented in Table 6, Table 7, Table 8, respectively. To further evaluate the robustness of the feature selection process, stability plots are constructed in Figure 2, Figure 3 for RF and *k*NN, respectively. These plots analyze the consistency of selected features across multiple iterations or subsets of data. Additionally, the boxplots shown in Figure 4, Figure 5 are utilized to visually represent the distribution of classification accuracy across different datasets for RF and *k*NN, respectively. These visual presentations highlight the consistency of the procedures across the datasets. Notably, the proposed method consistently outperforms the other methods in majority of the cases, as evidenced by the stability plots and boxplots.

**Table 6:** Results calculated for the top-ranked *p*^⁎^ feature selected by the proposed method and other state-of-the-art procedures using the *D*_5_ dataset.

Metric	$p^{⁎}$	RF								*k*NN							
		ROWSU	Fish	Wilc	SNR	POS	MRMR	WSNR	RFish	ROWSU	Fish	Wilc	SNR	POS	MRMR	WSNR	RFish
Accuracy	5	0.913	0.902	0.789	0.794	0.922	0.909	0.899	**0.935**	**0.895**	0.886	0.768	0.771	0.879	0.894	0.888	0.843
	10	0.927	0.915	0.809	0.807	0.936	0.915	0.914	**0.947**	0.824	0.906	0.777	0.778	0.898	0.898	**0.909**	0.839
	15	**0.935**	0.926	0.829	0.830	**0.935**	0.927	0.924	**0.935**	0.838	**0.920**	0.793	0.798	0.911	0.914	0.914	0.844
	20	**0.939**	0.927	0.826	0.840	0.938	0.930	0.922	0.930	0.867	**0.923**	0.787	0.798	0.919	0.916	0.914	0.842
	25	**0.939**	0.926	0.825	0.832	0.928	0.931	0.924	0.927	0.864	**0.923**	0.789	0.783	0.911	0.920	**0.923**	0.868
	30	**0.945**	0.935	0.845	0.838	0.943	0.940	0.925	0.916	0.883	0.923	0.796	0.800	0.920	**0.927**	0.921	0.875

Sensitivity	5	0.751	0.696	0.211	0.236	0.776	0.690	0.691	**0.858**	**0.816**	0.627	0.221	0.254	0.733	0.642	0.665	0.757
	10	**0.861**	0.718	0.227	0.223	0.805	0.709	0.719	0.801	**0.772**	0.696	0.275	0.235	0.762	0.659	0.719	0.789
	15	**0.810**	0.768	0.230	0.254	0.798	0.759	0.748	0.809	0.775	0.727	0.261	0.254	**0.838**	0.675	0.731	0.817
	20	**0.815**	0.765	0.235	0.274	0.803	0.776	0.751	0.795	0.774	0.746	0.249	0.292	**0.823**	0.693	0.740	0.811
	25	**0.812**	0.763	0.247	0.284	0.786	0.791	0.757	0.805	0.788	0.747	0.290	0.264	**0.831**	0.703	0.740	0.819
	30	0.808	0.755	0.271	0.252	0.817	0.792	0.770	**0.856**	0.776	0.720	0.266	0.294	**0.838**	0.703	0.734	0.798

F_1_-Score	5	0.855	0.689	0.526	0.731	0.777	0.748	0.803	**0.867**	0.782	0.667	0.402	0.727	0.693	0.644	**0.862**	0.803
	10	**0.878**	0.776	0.381	0.847	0.78	0.785	0.844	0.851	**0.829**	0.733	0.428	0.824	0.734	0.712	0.824	0.814
	15	**0.918**	0.808	0.401	0.903	0.838	0.819	0.847	**0.918**	0.698	0.782	0.353	0.816	0.784	0.765	**0.909**	0.847
	20	**0.923**	0.832	0.396	0.851	0.815	0.809	0.849	0.908	0.744	0.757	0.391	0.809	0.719	0.755	**0.891**	0.849
	25	**0.935**	0.756	0.493	0.817	0.792	0.757	0.863	0.896	0.620	0.760	0.418	0.847	0.732	**0.885**	0.848	0.863
	30	0.909	0.815	0.582	0.856	0.832	0.827	0.872	**0.915**	0.695	0.807	0.420	0.850	0.794	**0.876**	0.817	0.872

**Table 7:** Results calculated for the top-ranked *p*^⁎^ feature selected by the proposed method and other state-of-the-art procedures using the *D*_6_ dataset.

Metric	$p^{⁎}$	RF								*k*NN							
		ROWSU	Fish	Wilc	SNR	POS	MRMR	WSNR	RFish	ROWSU	Fish	Wilc	SNR	POS	MRMR	WSNR	RFish
Accuracy	5	0.734	0.734	0.699	0.687	0.732	0.739	0.732	**0.745**	0.694	0.766	0.669	0.677	0.683	0.777	0.773	**0.798**
	10	0.777	0.784	0.722	0.723	0.758	0.800	0.772	**0.806**	0.740	0.811	0.699	0.730	0.695	**0.833**	0.804	0.828
	15	**0.782**	**0.782**	0.738	0.752	0.752	**0.782**	0.775	0.780	0.758	0.805	0.713	0.702	0.717	0.821	0.818	**0.823**
	20	**0.781**	0.780	0.722	0.741	0.738	0.779	0.778	0.773	0.759	0.819	0.668	0.721	0.680	0.814	0.807	**0.836**
	25	0.797	0.789	0.755	0.729	0.745	0.801	0.780	**0.805**	0.768	0.824	0.692	0.703	0.714	0.820	0.812	**0.848**
	30	**0.777**	0.764	0.757	0.746	0.746	0.774	0.764	0.771	0.748	0.799	0.719	0.701	0.720	0.821	0.814	**0.834**

Sensitivity	5	0.343	0.340	0.220	0.179	0.282	0.341	0.330	**0.357**	0.413	0.393	0.193	0.207	0.251	0.400	0.411	**0.420**
	10	**0.476**	0.458	0.163	0.154	0.383	0.420	0.375	0.448	**0.531**	0.500	0.265	0.290	0.243	0.527	0.514	0.451
	15	0.405	0.376	0.136	0.161	0.255	0.349	0.397	**0.438**	**0.533**	0.456	0.321	0.278	0.164	0.504	0.525	0.449
	20	**0.500**	0.392	0.179	0.199	0.285	0.396	0.368	0.469	**0.672**	0.480	0.290	0.345	0.222	0.506	0.523	0.630
	25	**0.511**	0.440	0.284	0.169	0.273	0.453	0.371	0.466	0.625	0.518	0.303	0.344	0.221	0.527	0.470	**0.629**
	30	**0.459**	0.357	0.170	0.170	0.216	0.361	0.421	0.494	0.646	0.441	0.337	0.358	0.152	0.487	0.528	**0.657**

F_1_-Score	5	0.599	0.509	0.600	0.597	0.557	0.527	**0.609**	0.604	**0.657**	0.594	0.534	0.654	0.528	0.637	0.652	0.654
	10	**0.638**	0.561	0.625	0.613	0.543	0.615	0.631	0.587	0.639	0.575	0.437	0.756	0.504	0.657	**0.804**	0.758
	15	**0.658**	0.647	0.603	0.638	0.548	0.682	0.628	0.605	0.685	0.710	0.605	0.805	0.492	0.709	**0.818**	0.774
	20	**0.665**	0.672	0.661	0.628	0.628	0.674	0.655	0.619	0.689	0.683	0.565	0.783	0.571	0.736	0.802	**0.805**
	25	0.639	0.610	0.551	0.653	**0.653**	0.652	0.617	0.611	0.614	0.638	0.571	0.763	0.461	0.699	0.721	**0.815**
	30	**0.662**	0.647	0.711	0.661	0.553	0.653	0.619	0.652	0.692	0.749	0.562	0.809	0.567	0.752	0.742	**0.829**

**Table 8:** Results calculated for the top-ranked *p*^⁎^ feature selected by the proposed method and other state-of-the-art procedures using the *D*_7_ dataset.

Metric	$p^{⁎}$	RF								*k*NN							
		ROWSU	Fish	Wilc	SNR	POS	MRMR	WSNR	RFish	ROWSU	Fish	Wilc	SNR	POS	MRMR	WSNR	RFish
Accuracy	5	**0.798**	0.781	0.744	0.750	0.786	0.765	0.782	0.780	**0.818**	0.804	0.727	0.739	0.803	0.806	0.814	0.763
	10	**0.801**	0.791	0.766	0.777	0.795	0.781	0.786	0.774	0.813	0.848	0.758	0.772	**0.851**	0.823	0.832	0.784
	15	**0.834**	0.808	0.778	0.783	0.816	0.806	0.786	0.769	**0.869**	0.854	0.764	0.771	0.859	0.833	0.846	0.799
	20	**0.826**	0.796	0.782	0.787	0.824	0.799	0.780	0.795	**0.863**	0.850	0.780	0.770	0.843	0.841	0.844	0.814
	25	**0.803**	0.790	0.758	0.773	0.795	0.783	0.770	0.791	**0.839**	0.836	0.761	0.770	0.819	0.823	0.835	0.808
	30	**0.803**	0.784	0.768	0.775	0.799	0.786	0.789	0.758	0.818	0.836	0.774	0.776	0.840	0.829	**0.866**	0.841

Sensitivity	5	**0.443**	0.340	0.137	0.164	0.283	0.309	0.432	0.427	**0.584**	0.419	0.116	0.169	0.423	0.438	0.545	0.549
	10	**0.494**	0.405	0.073	0.129	0.397	0.326	0.362	0.361	**0.673**	0.569	0.151	0.224	0.511	0.497	0.544	0.551
	15	**0.567**	0.405	0.127	0.122	0.494	0.397	0.379	0.369	**0.773**	0.570	0.235	0.247	0.566	0.559	0.643	0.658
	20	**0.600**	0.353	0.100	0.102	0.424	0.363	0.322	0.336	**0.768**	0.576	0.303	0.172	0.440	0.604	0.569	0.599
	25	**0.476**	0.372	0.061	0.085	0.386	0.333	0.324	0.385	**0.734**	0.547	0.197	0.234	0.378	0.490	0.593	0.601
	30	**0.515**	0.389	0.090	0.079	0.393	0.391	0.397	0.412	**0.738**	0.565	0.281	0.209	0.478	0.559	0.687	0.657

F_1_-Score	5	**0.674**	0.600	0.667	0.660	0.657	0.569	0.650	0.615	0.605	0.620	0.579	**0.744**	0.731	0.654	0.642	0.657
	10	**0.678**	0.658	0.513	0.665	0.644	0.580	0.664	0.643	0.662	**0.737**	0.522	0.728	0.711	0.654	0.693	0.714
	15	**0.715**	0.711	0.698	0.676	0.710	0.618	0.707	0.709	0.715	**0.736**	0.671	0.698	0.681	0.645	0.705	0.731
	20	**0.758**	0.606	0.517	0.603	0.721	0.685	0.746	0.715	0.616	0.627	0.526	0.728	**0.736**	0.672	0.732	0.726
	25	**0.779**	0.738	0.562	0.700	0.741	0.729	0.753	0.726	**0.738**	0.710	0.622	0.731	0.683	0.712	0.719	0.733
	30	0.683	0.646	0.642	0.762	0.679	0.570	0.734	0.667	**0.772**	0.738	0.671	0.766	0.711	0.697	0.727	0.714

## 5 Conclusion

This work proposed a novel features selection method called robust weighted score for unbalanced data (ROWSU). It consisted of a sequence of steps to achieve the desired results where existing methods perform poorly in the presence of class-imbalanced problem. First, it balanced the dataset using the observations in the minority class. Second, ROWSU identified a minimum subset of the most discriminative features by using a greedy search approach in conjunction with a novel robust weighted score. The proposed method has given promising results when the class distribution of the dataset is extremely skewed. The proposed method is compared with state-of-the-art procedures on 7 benchmark datasets. The proposed ROWSU method outperformed the existing techniques in the majority of the cases. The performance of the ROWSU method and other feature selection methods are evaluated through the random forest (RF) and *k* nearest neighbors (*k*NN) classifiers. Classification accuracy, sensitivity and F_1_-score have been used as evaluation metrics. Moreover, stability plots of the results are constructed in the paper for different numbers of features which show the consistency of the proposed method. For a better understanding of the performance, boxplots of the results for the top 10 features have also been constructed. The results given in the paper demonstrated that the proposed method can effectively solve the class-imbalanced problem and discard non-informative and redundant genes.

## CRediT authorship contribution statement

**Zardad Khan:** Writing – original draft, Visualization, Supervision, Software, Data curation, Conceptualization. **Amjad Ali:** Writing – original draft, Visualization, Software, Formal analysis, Data curation, Conceptualization. **Saeed Aldahmani:** Writing – original draft, Methodology, Conceptualization.

## Declaration of Competing Interest

The authors declare that they have no known competing financial interests or personal relationships that could have appeared to influence the work reported in this paper.

## Data Availability

Data included in this study is referenced in the article.
